# Safety and Tolerability of Inhaled Aztreonam in Children and Adolescents: A Systematic Review and Meta-Analysis

**DOI:** 10.3390/arm93050038

**Published:** 2025-09-26

**Authors:** Valmir N. Rastely-Junior, Hosanea S. N. Rocha, Mitermayer G. Reis

**Affiliations:** 1 Faculdade de Medicina da Bahia, Universidade Federal da Bahia (UFBA), Salvador 40026-010, Brazil; valmir.j.rastely@gmail.com; 2 Departamento de Medicina, Escola Bahiana de Medicina e Saúde Pública (EBMSP), Salvador 40290-000, Brazil; 3 Instituto Gonçalo Moniz, Fundação Oswaldo Cruz (Fiocruz), Salvador 40296-710, Brazil

**Keywords:** aztreonam, adverse effects, safety, inhaled antibiotics, pediatrics, cystic fibrosis, *Pseudomonas aeruginosa*, pediatric safety, meta-analysis, systematic review

## Abstract

**Highlights:**

**What are the main findings?**

- Comparative analyses showed that inhaled aztreonam did not increase the risk of common adverse events (cough, dyspnea, fever, headache) compared with placebo or other inhaled antibiotics; however, serious grade 3/4 respiratory disorders were more frequent.
- A reduced risk of pulmonary function decline was observed in patients receiving inhaled aztreonam, with most adverse events being mild, manageable, and reversible.

**What is the implication of the main finding?**

- The overall tolerability and safety profiles support the use of inhaled aztreonam in children and adolescents with cystic fibrosis, provided that clinicians monitor for severe respiratory reactions and rare hepatotoxic effects.
- Future trials with larger sample sizes and longer follow-up should evaluate dosing strategies and investigate rare adverse events to refine risk–benefit assessments in pediatric populations.

**Abstract:**

Respiratory infections and chronic lung disease are major contributors to morbidity in children. Aztreonam lysine for inhalation (AZLI) delivers high local antibiotic concentrations while limiting systemic exposure; however, its safety in younger patients remains uncertain. This systematic review and meta-analysis searched MEDLINE, CENTRAL, and Google Scholar for randomized and observational studies reporting adverse events in children and adolescents (≤18 years) receiving AZLI, with no date limit. Fourteen studies were included. Most studies were moderate-to-high quality. Comparative analysis showed no clinically relevant increase in common adverse events relative to placebo or other inhaled antibiotics. The pooled relative risk for severe respiratory disorders (grade 3/4) was 1.65 (95% CI 1.07–2.57), suggesting a higher incidence of serious respiratory events, while a protective effect against decline in pulmonary function was observed (RR 0.70, 95% CI 0.54–0.90). Adverse events were generally mild; serious adverse events and hospitalizations were infrequent and comparable between groups. Cumulative prevalence estimates indicated that respiratory irritation occurred in 10–25% of patients, whereas systemic effects were uncommon. Overall, AZLI appears to have an acceptable tolerability and safety profile in children and adolescents, though careful monitoring is warranted, especially for severe respiratory events.

## 1. Introduction

Respiratory infections remain a leading challenge in pediatric health worldwide. Acute lower respiratory tract infections, including bacterial and viral pneumonias, are the single largest infectious cause of death in children, killing about 740,180 children under five years of age in 2019, and accounting for 14% of all under-five deaths [1]. The burden of respiratory disease extends beyond acute infections. Chronic conditions such as bronchiectasis, primary ciliary dyskinesia, and cystic fibrosis (CF) predispose children to persistent lower-airway infections that are difficult to treat. These disorders often harbor multidrug-resistant bacteria—including *Pseudomonas aeruginosa*, methicillin-resistant *Staphylococcus aureus, Burkholderia* spp., and non-tuberculous mycobacteria—necessitating intensive antimicrobial strategies [2]. Among chronic disorders, CF is the most common inherited condition associated with recurring endobronchial infections. Registry data indicate that there are roughly 30,000 people with CF in the United States and 70,000 worldwide [3]. Pulmonary complications are the major cause of death in CF, accounting for nearly 60% of CF-related mortality [3]. *P. aeruginosa* colonization becomes increasingly common with age: in a multinational cohort, about half of children with CF acquired *P. aeruginosa* by 5.1 years of age, and 25% developed chronic colonization by 14.7 years [4]. Historical reports suggest that about 80% of individuals are infected by the age of 18 years [5]. Early infection is associated with a more rapid decline in lung function and worse survival [5]. Even with eradication strategies, persistent *P. aeruginosa* infection remains a significant cause of morbidity, highlighting the need for effective and safe inhaled therapies.

Systemic antipseudomonal antibiotics enhance outcomes but are constrained by toxicity and the requirement for intravenous access. Inhaled delivery gets a lot of the drug to the site of infection while minimizing how much of it gets into the rest of the body [6]. Aztreonam lysine for inhalation (AZLI) is a synthetic monobactam whose activity is mediated by inhibition of bacterial cell wall assembly, and works against gram-negative bacteria, such as *P*. *aeruginosa*. It binds with high affinity to penicillin-binding protein-3 (PBP3) in bacteria, blocking the transpeptidase step of peptidoglycan cross-linking. PBP3 inhibition triggers autolytic enzymes and membrane rupture, resulting in bactericidal effects [6].

The U.S. Food and Drug Administration (FDA) approved AZLI in 2010 to help CF patients with chronic *P*. *aeruginosa* infection breathe better [7]. After that, guideline committees included AZLI in standard care. According to the Cystic Fibrosis Foundation (CFF), AZLI should be given to those over 6 years old who have mild, moderate, or severe lung illness and a chronic *P*. *aeruginosa* infection to improve lung function and quality of life [8]. Subsequent trials showed that long-term alternate-month AZLI therapy maintained lung function and quality-of-life benefits without increasing resistance, and increasing the time to need antibiotics [2,9]. These data established AZLI as a key option for suppressing chronic *P. aeruginosa* infections in pediatric CF.

Inhaled antibiotics generally have favorable safety profiles compared with systemic therapy, yet respiratory irritation and systemic effects remain concerns. Across clinical trials, AZLI has been well tolerated, but the most frequently reported adverse reactions include cough (32–35%), headache (6–11%), nasal congestion (7–10%), rhinorrhea (~7%), and bronchospasm (6–10%) [2]. Occasional bronchoconstriction has prompted recommendations for a monitored test dose in patients with severe lung disease. Existing studies, however, have several limitations: many were conducted before widespread use of CF transmembrane conductance regulator (CFTR) modulators, enrolled relatively small numbers of children and adolescents, and prioritized efficacy over systematic adverse-event collection; evidence in very young children is sparse, and one randomized trial in patients colonized with *Burkholderia cepacia* found no improvement in lung function despite similar adverse-event rates [2]. Given that CFTR modulators have changed the course of cystic fibrosis, inhaled antibiotics such as AZLI warrant a fresh safety and relevance evaluation. Furthermore, post-marketing pharmacovigilance has highlighted unexpected safety signals. A recent disproportionality analysis of the U.S. Food and Drug Administration Adverse Event Reporting System, including 11,627 reports listing aztreonam as the primary suspect. It identified 127 preferred-term safety signals. Rare but serious events were reported, such as cholestatic liver injury, hypoprothrombinemia, hemoptysis, pulmonary hemorrhage, drug reaction with eosinophilia and systemic symptoms (DRESS), and acute generalized exanthematous pustulosis. For AZLI specifically, the median time to onset of adverse events was approximately one year [10]. Such evidence would inform clinicians, caregivers, and guideline committees about the balance between benefits and harms, support shared decision-making, and identify knowledge gaps for future research.

The lack of a specific quantitative synthesis of AZLI safety in children and adolescents presents difficulties for clinicians and policymakers. Current meta-analyses generally aggregate data across extensive age ranges or prioritize efficacy outcomes, resulting in ambiguity on the incidence and severity of adverse events in younger patients. A systematic review and meta-analysis of adverse events linked to inhaled aztreonam can aggregate data from trials and observational studies to ascertain the prevalence of particular side effects and evaluate risks related to control therapy. Such evidence would elucidate the equilibrium between benefits and detriments, facilitate collaborative decision-making, and pinpoint knowledge deficiencies for subsequent research endeavors.

The primary objective of this review is to quantify the comparative risk of adverse events associated with inhaled aztreonam in children and adolescents. Secondary objectives include quantifying the pooled prevalence of adverse events associated with AZLI in the pediatric population. The primary research inquiries are as follows:

1. Do the risks of specific adverse events differ between AZLI and placebo or other inhaled antibiotics?
2. What is the pooled prevalence of adverse outcomes among children and adolescents receiving AZLI?

## 2. Materials and Methods

### 2.1. Register and Guidelines

This systematic review was designed in accordance with the PRISMA 2020 statement guidelines [11] and was prospectively registered in PROSPERO under registration number CRD420251084278. The study commenced on 2 July 2025, with completion anticipated for 2 August 2025.

### 2.2. Eligibility Criteria (PICOS)

Eligible studies evaluated children and adolescents (<18 years at treatment initiation) of both sexes, all pediatric conditions, country, language, or care setting (outpatient or inpatient). We included randomized trials and observational studies that investigated AZLI in any formulation, dose, duration, or device, whether administered alone or in combination with other treatments. All comparators were accepted (placebo, standard therapy, other inhaled antimicrobials, or comparisons between regimens). Preclinical studies (animal or in vitro models) were excluded.

### 2.3. Information Sources

Search was conducted in the MEDLINE database (via PubMed) and CENTRAL (Cochrane Central Register of Controlled Trials), with no language or date restrictions. For grey literature, we consulted Google Scholar and performed manual searches of article reference lists and citations (snowballing). We also examined clinical trial registries when available. All records were imported into Zotero for organization and deduplication. Title/abstract and full-text screening was conducted in Rayyan and in the Excel sheets [12]. We intentionally excluded EMBASE because of the overlap with the main databases and did not yield any unique eligible studies beyond those already identified.

### 2.4. Search Strategy

We employed a sensitive search strategy based on keywords, synonyms, and MeSH terms, covering three domains: drug (aztreonam and its synonyms), route of administration (inhalation/nebulization/aerosol), and pediatric population. The complete PubMed search string was as follows:

(“aztreonam” OR “Azthreonam” OR “Az-threonam” OR “Azactam” OR “SQ-26,776” OR “SQ 26,776” OR “SQ26,776” OR “Aztreon *” OR “azonam” OR “primbactam” OR “nebactam”) AND (“inhal*” OR “nebul*” OR “aerosol*” OR “atomaz*” OR “vapor*” OR “Nebulizers and Vaporizers”[Mesh] OR “Inhalation”[Mesh] OR “Administration, Inhalation”[Mesh]) AND ((infan* OR newborn* OR new-born * OR neonat* OR baby* OR babies OR toddler* OR minors OR boy OR boys OR boyhood OR girl * OR twin * OR kid OR kids OR child* OR stepchild OR step-child OR preschool OR schoolchild OR pediatrics[mh] OR pediatric* OR paediatric* OR peadiatric* OR school*[tiab] OR premature * OR preterm OR Child[Mesh] OR Infant[Mesh])).

The syntax was adapted for other databases (CENTRAL, Google Scholar).

### 2.5. Study Selection Process

Study selection was performed in two stages. During the screening of titles and abstracts, two reviewers independently and blindly assessed each record; disagreements were discussed and, when necessary, resolved with the involvement of a third reviewer. The same approach was applied to the full-text review, with standardized documentation of exclusion reasons in a PRISMA flow diagram: population outside the target age range, ineligible intervention, lack of safety and tolerability data, not an original article, not a clinical study, and unavailability of abstract and/or full text.

### 2.6. Data Extraction

Two reviewers independently extracted data using a standardized form that had been previously piloted. For each study, we collected information on identification (author, year of publication, study period), country, study design, setting (outpatient or inpatient), sample size, age (mean or median and range), diagnosis, and eligibility criteria. We detailed the intervention (dose, frequency—for example, BID/TID, inhalation device, treatment duration, total study period, and follow-up) and the comparator (type of control, if present). Discrepancies were resolved by consensus or, if needed, by a third reviewer.

### 2.7. Risk of Bias Assessment

Risk of bias was evaluated using the Cochrane RoB 2 tool for randomized clinical trials [13]. Two independent reviewers applied the tool and discussed the results; persistent disagreements were resolved by consensus. These judgments were integrated into the interpretation of results and the assessment of certainty of evidence using GRADE. “Traffic light/robvis” diagrams will be used to visually present the results.

### 2.8. Comparative Meta-Analysis

When a comparator group was present, we calculated the relative risk (RR) of adverse events using random-effects models by inverse variance. To handle zero-event data, a continuity correction of 0.5 was applied only to zero cells, and studies with double zeros were included [14]. Between-study heterogeneity was quantified using τ^2^ and the I^2^ statistic. The between-study variance (τ^2^) was estimated using restricted maximum likelihood (REML) [15], and confidence intervals were calculated using the Hartung–Knapp–Sidik–Jonkman (HKSJ) method [16]. From the pooled RR and 95% prediction interval (PI), we derived the corresponding risk in the aztreonam group (assumed risk). Tests for publication bias were conducted when there were ten or more studies.

### 2.9. Proportional Meta-Analysis

For studies with and without comparator groups, cumulative prevalence was calculated, and the proportion of participants experiencing at least one adverse event was synthesized using random-effects models with τ^2^ using REML on the Freeman–Tukey (double arcsine) scale with back-transformation and 95% prediction interval (PI). Confidence intervals for each study were obtained using the exact binomial (Clopper–Pearson) method; the pooled confidence interval was calculated using the HKSJ method [17].

### 2.10. Software

All analyses were conducted using R (RStudio 2025.05.01).

### 2.11. Certainty of Evidence (GRADE)

The certainty of evidence was evaluated for each outcome using the GRADE approach [18]. For comparative outcomes, the summary of Findings tables presents the RR and its 95% CI (HKSJ), the assumed risk (median of risks in the control group), the corresponding risk in the aztreonam group, the absolute risk increase, when RR > 1 (or indication of benefit when RR < 1). For proportional meta-analyses, we report the pooled prevalence with 95% CI. The GRADE rating starts at “high” for randomized trials and “low” for observational studies, including single-arm studies. The studies were downgraded for risk of bias, inconsistency, indirectness, imprecision, or publication bias; and upgrades when applicable (large effect, dose-response gradient, or residual confounding opposing the observed effect) [19].

## 3. Results

### 3.1. Selection and Inclusion of Records in the Systematic Review

**Figure A1:**
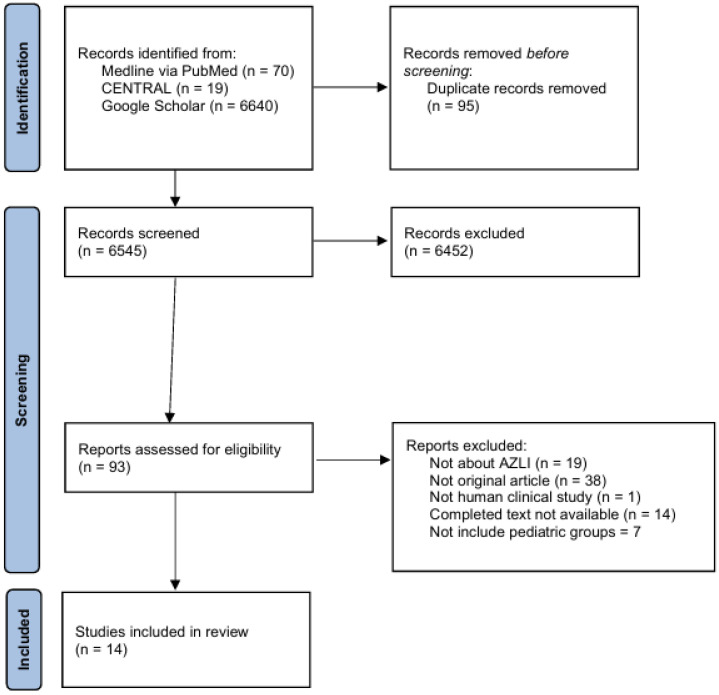
PRISMA flow diagram of study identification, screening, and inclusion for the systematic review of inhaled aztreonam tolerability and safety in pediatric populations. Note: AZLI: Aztreonam lysine for inhalation; CENTRAL: Cochrane Central Register of Controlled Trials; *n*: Number of records or studies. This PRISMA flow diagram details the multi-stage process of identifying, screening, and including studies for this systematic review, following the PRISMA 2020 guidelines. “Reports excluded” refers to studies excluded after full-text assessment, with detailed reasons listed. “Duplicate records removed” denotes deduplication across databases. Screening was conducted on titles and abstracts, followed by full-text assessment for eligibility. Only studies including pediatric populations were retained.

The systematic search of Medline through PubMed, CENTRAL, and Google Scholar yielded 6545 entries, excluding duplicates, of which 93 articles were evaluated in full text. Figure A1 shows that 14 studies were included for qualitative analysis after the eligibility criteria were applied. These studies were published between 1987 and 2023, with more published in the last 15 years. This shows that there is more interest in the therapeutic effects of inhaled aztreonam in children [9,20,21,22,23,24,25,26,27,28,29,30,31,32].

As shown in Table 1, the conduct period for the included studies ranged from 2003 to 2021. Methodological robustness was demonstrated by the predominance of randomized clinical trials (64% of included studies), encompassing phase 2 and 3 trials, double-blind, placebo-controlled designs, as well as open-label and dose-escalation studies [9,21,26,27,28,29,30,31]. Observational studies accounted for 29% of the sample and included both open single-arm designs and studies with active comparators [20,22,24,32]. Additionally, a post hoc analysis of a multicenter trial was included, further broadening the methodological spectrum [31].

Most studies were conducted in the United States, with six single-center studies in that country [9,20,21,27,28,29]. Four additional multicenter studies involved intercontinental collaborations between the USA, Canada, Australia, and New Zealand, while three studies were conducted jointly in Europe and the USA. One study did not report the geographic location of its conduct [30].

An analysis of the 14 included studies revealed significant diversity in the age profile of participants assessed for the safety of inhaled aztreonam. While most trials included both adults and children, three studies [22,30,31] focused exclusively on the pediatric population, underscoring the focus and relevance of the results for children and adolescents with cystic fibrosis (CF). The age range of patients treated with inhaled aztreonam was broad, from infants as young as 3 months to young adults and elderly individuals, especially in studies with mixed samples. Nonetheless, most participants were aged between 3 months and less than 18 years, with mean ages in pediatric studies primarily ranging from 6 to 15 years [21,24,27,29,30]. This age representation guarantees that the safety facts provided herein are directly relevant to pediatric and adolescent demographics. Concerning sex, the study samples exhibited sufficient female representation. Only two studies [25,29] did not specify the sex of participants. The clinical characteristics of the studied populations were also highly heterogeneous. All studies involved children and adolescents with a confirmed diagnosis of cystic fibrosis (CF). However, the clinical criteria differed: certain trials concentrated on patients with chronic Pseudomonas aeruginosa infection, while others encompassed children with recent or initial infection, in addition to cases with chronic *Burkholderia* infection. There was also a range of pulmonary function criteria, including cases with stable illness and moderate to severe lung disease. This helped evaluate the safety of inhaled aztreonam in varied clinical settings for children with CF.

Some studies utilized single-arm or cohort designs lacking a control group [19,21,24,29,31], whilst others integrated a placebo with regular medicines, illustrating the intricacies of clinical practice [20]. Most trials utilized placebo groups, typically consisting of lactose or saline solutions, as comparators [8,22,24,25,26,27]. Some studies also looked at aztreonam directly against other routinely used antibiotics, like inhaled tobramycin [23] and combinations of azlocillin and intravenous tobramycin [28]. Other research chooses to look at different doses or concentrations of aztreonam, which makes it possible to see how safe it is in a variety of situations.

Analysis of studies on inhaled aztreonam in children and adolescents with cystic fibrosis revealed that the predominant regimen was 75 mg three times daily, typically administered in 28-day cycles followed by rest periods. This design was used in most trials [20,21,22,23,24,25,27,29], which made it easy to compare the results. Some studies tried different doses and lengths of time; however, they did not change the fact that the 28-day cycle was the most common. This cycle was valuable for testing the safety and effectiveness of the intervention in varied clinical situations.

The assessed clinical indications were diverse, including the eradication or early prevention of Pseudomonas aeruginosa infection, maintenance and bacterial suppression, and the management of acute exacerbations. The 75 mg TID regimen for 28 days demonstrated efficacy in eliminating the germs and reducing respiratory symptoms during investigations focused on eradication. In prolonged therapeutic contexts, inhaled aztreonam enhanced quality of life, lung function, and diminished exacerbations, while exhibiting a safety profile akin to that of a placebo. Investigations into acute exacerbations and pharmacokinetics indicated that the medication was well-tolerated, even when administered concurrently with other pharmaceuticals.

The studies included used inhaled administration, mostly with electronic equipment like the eFlow^®^/Altera^®^ nebulizer (**PARI Pharma GmbH Gräfelfing, Germany)**, which may be used by people of all ages and in diverse clinical settings [8,20,21,22,24,25,27,29,31]. Protocols sometimes suggested the use of bronchodilators before giving aztreonam, and self-administration under supervision was common. The majority of investigations indicated the utilization of various concomitant medications, encompassing inhaled antibiotics (such as tobramycin, colistin), azithromycin, mucolytics, bronchodilators, hypertonic saline solutions, corticosteroids, pancreatic enzymes, and vitamins [8,20,24,25,26,28].

There was a lot of variety in how long the trials on inhaled aztreonam lasted, with follow-up periods ranging from 13 days to five years. Furthermore, the diverse time points utilized for outcome collecting and analysis demonstrate rigorous scientific practices, encompassing evaluations throughout various therapy phases and after many cycles. Studies included both early monitoring of adverse events—through assessments completed hours after administration—and long-term follow-up, with regular visits lasting up to five years, enhancing the thoroughness of safety and efficacy analyses across clinical settings.

Outcomes analyzed were comprehensive, including microbiological eradication, changes in lung function (FEV1), respiratory symptoms, and exacerbation rates, as well as tolerability, laboratory safety, and bacterial resistance. This multiplicity of outcomes reflects a balanced concern for both efficacy and safety.

As shown in Table A1, the risk of bias analysis, conducted using the RoB 2 tool, revealed that only four studies were classified as having low risk of bias, demonstrating methodological robustness primarily through adequate randomization and transparent outcome reporting [4,20,22,25]. These studies serve as references for the most reliable interpretation of inhaled aztreonam safety data in children and adolescents. On the other hand, the majority of included studies—nine of the fourteen analyzed—were classified as high risk of bias [8,19,21,23,24,25,27,31]. The main limitations identified related to lack of blinding, substantial participant losses during follow-up, absence of intention-to-treat analysis, and insufficient or selective reporting of evaluated outcomes. Notably, in domains relating to intervention adherence, handling of missing data, and selection of reported outcomes, several studies were classified as high risk or “some concerns,” further compromising the reliability of the findings [12,21,24,28].

### 3.2. Comparative Meta-Analysis of Adverse Events

The comparative meta-analysis reveals that the administration of inhaled aztreonam in children and adolescents does not correlate with a clinically significant rise in the majority of prevalent adverse events when compared to controls, as illustrated in Table 2. In high-frequency respiratory outcomes, productive sputum exhibited a relative risk (RR) of 0.83 (95% CI 0.67–1.02; I^2^ 2.3%), sputum congestion RR 0.84 (0.54–1.31; I^2^ 0%), rales/crackles RR 0.84 (0.60–1.20; I^2^ 0%), and dyspnea RR 0.76 (0.51–1.13; I^2^ 0%). The narrow confidence intervals and limited heterogeneity offer moderate evidence of the absence of increased harm.

A major exception was noted for severe (grade 3/4) respiratory problems, where aztreonam markedly elevated the risk (RR 1.65; 1.07–2.57; I^2^ 0%); however, the certainty is low due to methodological constraints and imprecision. From a functional standpoint, the medication was linked to a reduced probability of deterioration in pulmonary function (PFT), with a relative risk of 0.70 (0.54–0.90; I^2^ 0%), indicating a strong and potentially clinically protective outcome.

Among systemic effects, fatigue (RR 0.71; 0.38–1.31; I^2^ 0%), pyrexia (RR 1.11; 0.82–1.50; I^2^ 0%), and headache (RR 1.03; 0.70–1.52; I^2^ 0%) exhibited no statistically significant increases. Regarding gastrointestinal signs, abdominal pain/tenderness exhibited a relative risk (RR) of 0.62 (0.15–2.63; I^2^ 63%), vomiting had an RR of 0.97 (0.48–1.96; single study), and diarrhea presented an RR of 0.36 (0.10–1.26; single study); dysgeusia demonstrated an RR of 4.65 (0.27–81.62; single study). The evidence for these outcomes is highly questionable, as it originates from a restricted number of studies or small samples, with confidence intervals covering significant effects in both directions.

Regarding laboratory abnormalities, namely hepatotoxicity, the relative risk (RR) was 4.00 (1.08–14.75), while for general laboratory abnormalities, the RR was 2.00 (0.85–4.73). Both findings were from a singular experiment with 22 patients, demonstrating no substantial heterogeneity; hence, these data necessitate careful interpretation.

**Figure A2:**
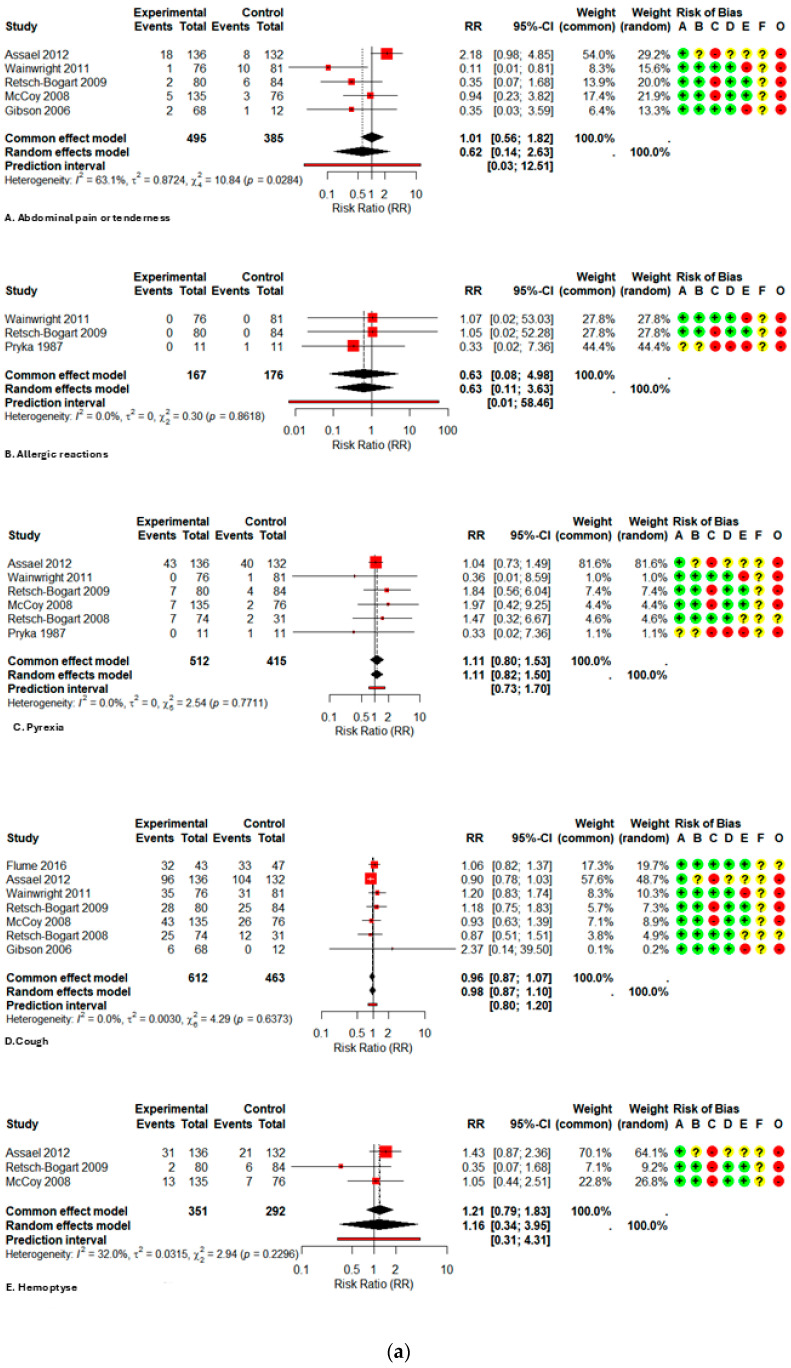

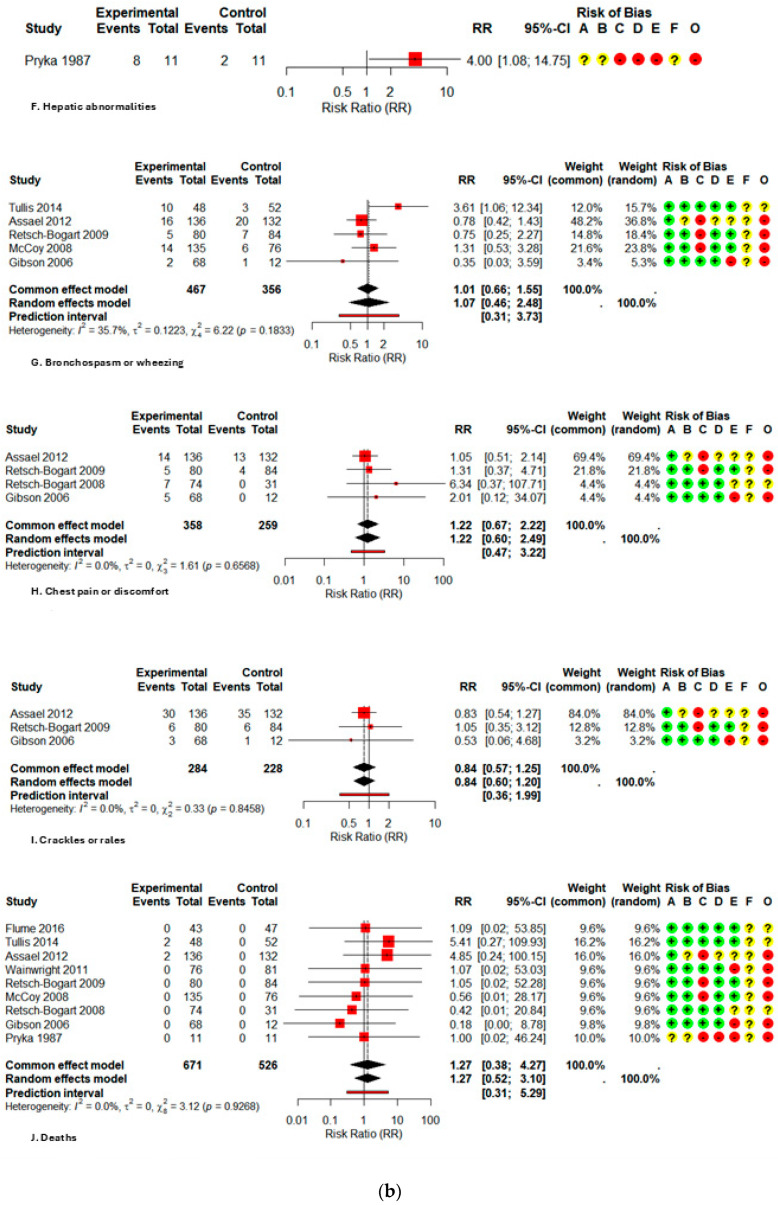

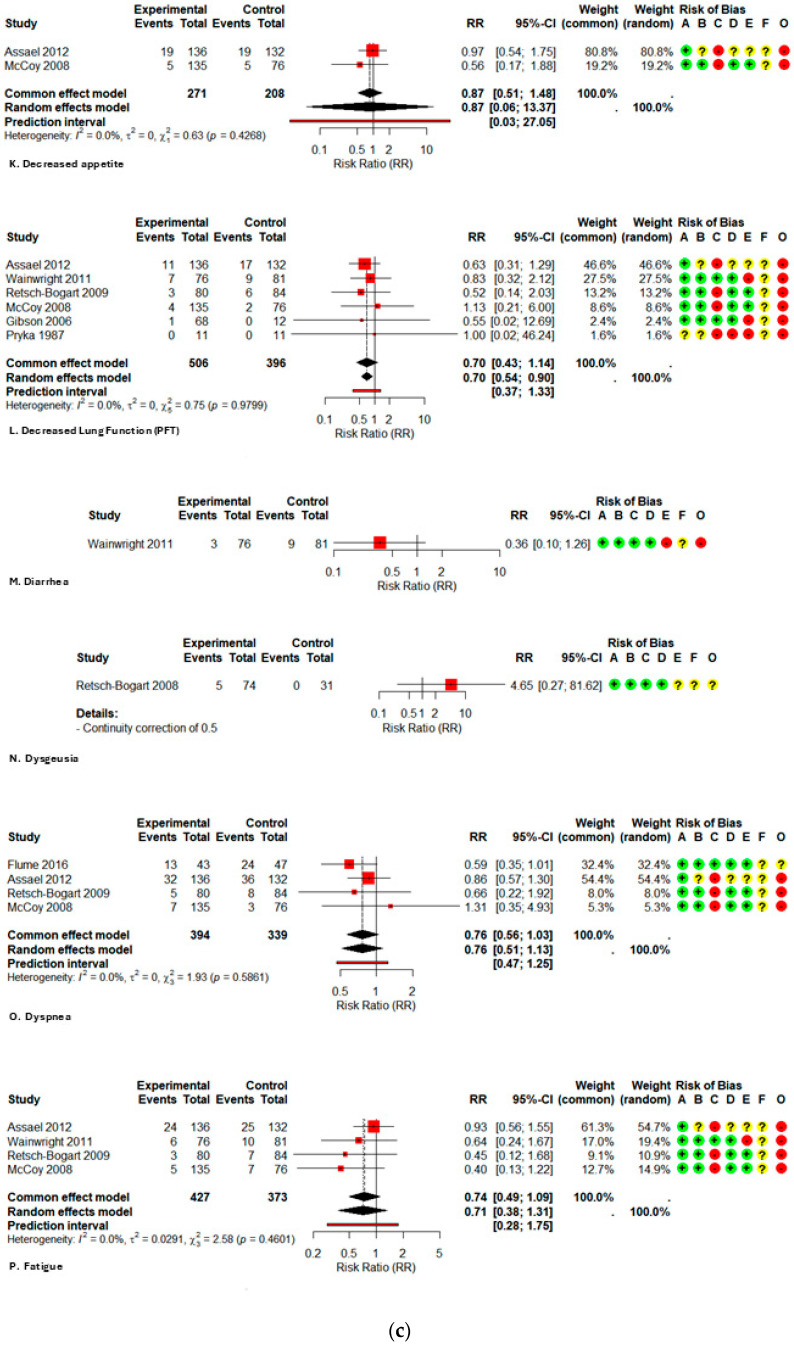

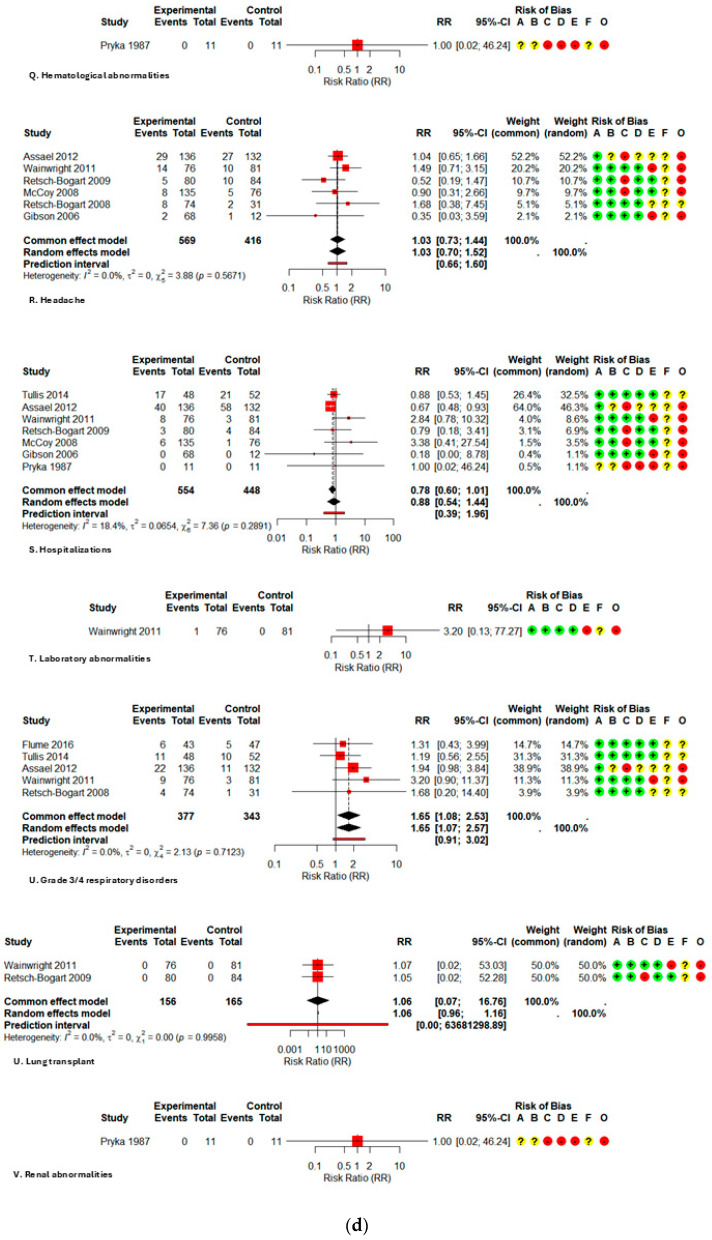

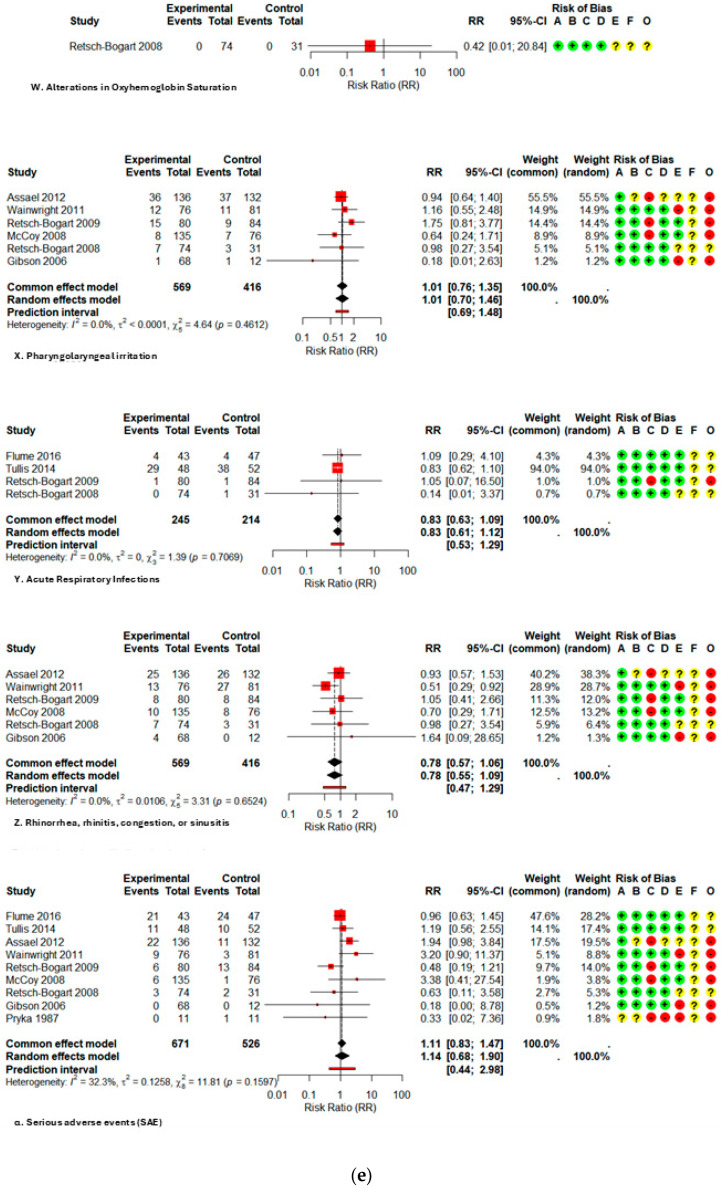

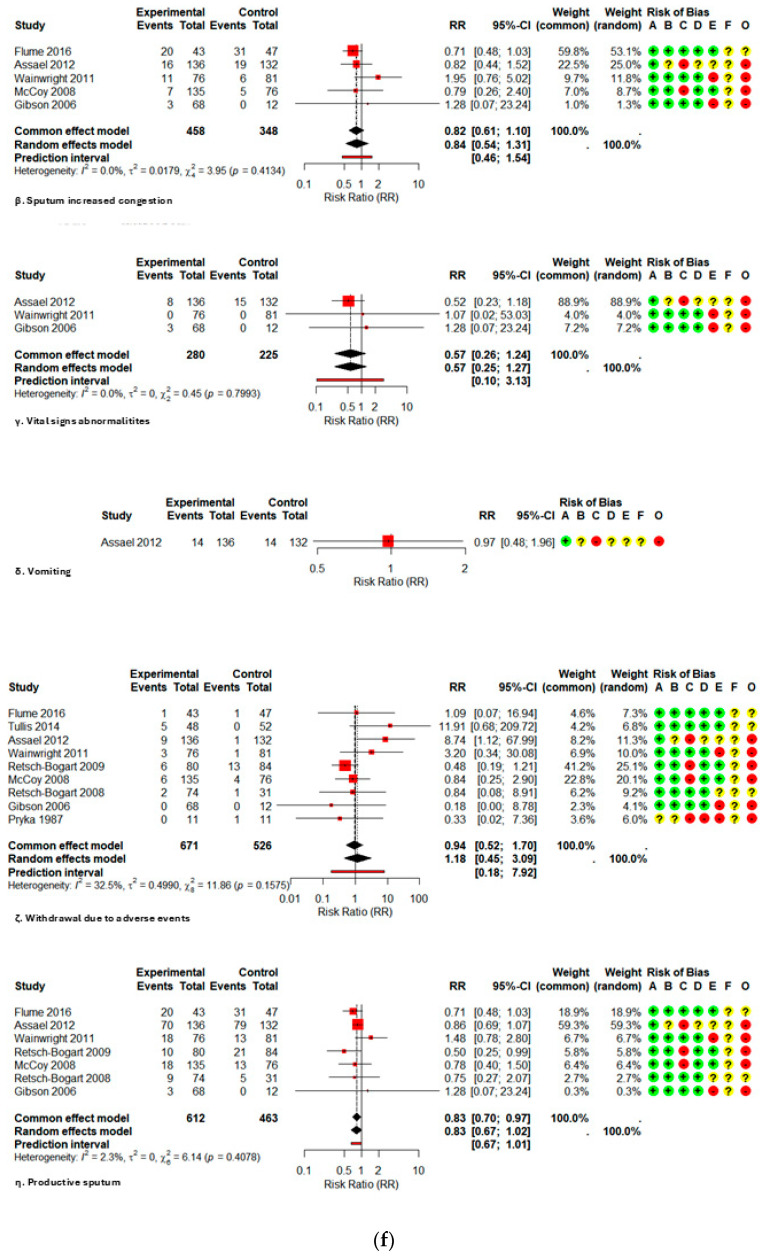
Forest plot of risk ratios for each adverse event with AZLI in children and adolescents: (**a**) abdominal pain or tenderness, allergic reactions, pyrexia, cough, hemoptysis; (**b**) hepatic abnormalities, bronchospasm or wheezing, chest pain or discomfort, crackles or rales, deaths; (**c**) decreased appetite, decreased lung function, diarrhea, dysgeusia, dyspnea, fatigue; (**d**) hematological abnormalities, headache, hospitalizations, laboratory abnormalities, grade ¾ respiratory disorders, lung transplant, renal abnormalities; (**e**) alterations in oxyhemoglobin saturation, pharyngolaryngeal irritation, acute respiratory infections, rhinorrhea, rhinitis, congestion, sinusitis, serious adverse events; (**f**) sputum increased congestion, vital signs abnormalities, vomiting, withdrawal due to adverse events, productive sputum. Abbreviations and Notes: AZLI: Aztreonam lysine for inhalation; RR: Risk ratio; CI: Confidence interval; I^2^: Percentage of total variation across studies due to heterogeneity; τ^2^: Between-study variance in random-effects meta-analysis; Q: Cochran’s heterogeneity statistic; *p*: *p*-value; A–F: Risk of bias domains (A: randomization, B: intervention deviations, C: missing data, D: outcome measurement, E: reporting, F: additional); O: Overall risk of bias. Risk ratios >1 suggest higher risk in the aztreonam group; <1 suggest lower risk. Weights reflect the contribution of each study to pooled analysis. All analyses are based on inverse variance methods. A continuity correction of 0.5 was applied to studies with zero events in both groups to allow meta-analysis [9,21,23,24,25,26,27,28,29].

Data on serious adverse events (SAEs) (RR 1.14; 0.68–1.90; I^2^ 32.3%), hospitalizations (RR 0.88; 0.54–1.44; I^2^ 18.4%), and mortality (RR 1.27; 0.52–3.10; I^2^ 0%) were infrequent and exhibited no significant differences between groups; however, the certainty is classified as low to very low due to the infrequency of outcomes and considerable imprecision. All forest plots are presented in Figure A2.

Owing to the limited number of papers used in this meta-analysis (*n* < 10), no formal assessment of publication bias was performed, utilizing funnel plots or statistical tests for asymmetry, as advised by the Cochrane Handbook [14]. Searches of grey literature and trial registries were conducted to mitigate the likelihood of publication bias, and the potential constraints associated with this bias were qualitatively analyzed.

### 3.3. Proportional Meta-Analysis of Adverse Events

For the cumulative prevalence meta-analyses of adverse events, as shown in Table A2, among respiratory outcomes, mild events such as productive sputum showed a pooled prevalence of 0.25 (95% CI 0.08–0.47; I^2^ 98.2%, Q 455.9, *p* < 0.0001), while sputum congestion presented 0.19 (0.03–0.43; I^2^ 97.0%). Among potentially severe events, bronchospasm/wheezing occurred in 0.10 of participants (0.02–0.21; I^2^ 93.3%), and grade 3/4 respiratory disorders reached 0.15 (0.06–0.27; I^2^ 94.7%). The high heterogeneity (I^2^ > 90% for all these outcomes) suggests the influence of clinical differences (e.g., age, baseline lung function) or methodological differences between studies.

Among systemic manifestations, fever/pyrexia was relatively common, with a prevalence of 10.9 (2.4–23.7; I^2^ 96.1%, Q 206.5, *p* < 0.0001). Fatigue and headache showed similar estimates—0.13 (0.01–0.34; I^2^ 96.6%) and 0.13 (0.05–0.24; I^2^ 92.3%), respectively—again with substantial variability. Gastrointestinal events such as abdominal pain (4%, 1–11; I^2^ 75%) and vomiting (5%, 3–8; I^2^ 90.8%) occurred in up to one in twenty patients, but with wide confidence intervals and marked heterogeneity, reflecting considerable uncertainty in the estimates.

Regarding severe complications, serious adverse events (SAEs) had a prevalence of 0.11 (0.03–0.23; I^2^ 96.1%, Q 285.7, *p* < 0.0001). Hospitalizations occurred with greater frequency, recorded at 15.3 (4.9–29.7; I^2^ 97.3%), although mortality remained infrequent at 0.02 (0.00–0.36) with I^2^ 0%. Although fatalities are infrequent, the substantial variety in serious adverse events and hospitalizations suggests potential methodological and clinical discrepancies, necessitating additional analyses (e.g., meta-regression) to ascertain causes of heterogeneity.

Laboratory abnormalities (1.7, 0–9.4; I^2^ 75.2%) and audiological abnormalities (1.7, 0–9.4; I^2^ 75.2%) exhibited borderline heterogeneity, indicating that a limited number of severe observations may distort the estimations. Extremely rare occurrences, such as lung transplantation (0.00, 0.00–0.001; I^2^ 0%) and allergic responses (0.00, 0.00–0.50; I^2^ 0%), remained nearly negligible; nonetheless, the data are highly questionable due to the restricted number of studies and patients.

## 4. Discussion

### 4.1. Overview of the Evidence Base

This review indicates that the most common adverse events (AEs) did not increase in a clinically relevant way with the use of inhaled aztreonam in children and adolescents. One important exception was observed for grade 3/4 respiratory disorders, for which risk increased with AZLI. On the other hand, the drug demonstrated a signal of protection against lung function decline: the significant reduction in the occurrence of pulmonary function deterioration (PFT) suggests a potential clinical benefit. Rare outcomes such as serious adverse events, hospitalizations, and mortality showed no statistical difference; however, the certainty of evidence is low due to the limited number of events. Laboratory abnormalities, especially hepatotoxicity, were evaluated in only one trial and suggested an increased risk, but the small sample size limits firm conclusions. Cumulative prevalence estimates revealed that mild respiratory events are common in the context of inhaled aztreonam. Severe events remained infrequent. Uncertainty remains high because confidence intervals are wide and inter-study heterogeneity is substantial.

### 4.2. Clinical Interpretation and Significance

From a practical standpoint, the data suggest that the safety profile of inhaled aztreonam is acceptable for most children and adolescents with CF when compared to placebo or reference therapies. The absence of a significant increase in events such as productive sputum, congestion, dyspnea, fatigue, and fever indicates that the local effects of the drug are predictable and generally manageable. The reduction in the risk of lung function decline suggests a potential clinical benefit, especially in patients with stable or declining pulmonary function. The observed increase in severe respiratory disorders, although based on few events, should be considered in the risk–benefit assessment: these conditions include exacerbations or bronchospasm that may require medical intervention. In clinical practice, treatment should be individualized, with respiratory monitoring particularly during the first weeks of use and in patients with advanced lung disease.

The high heterogeneity observed in many outcomes likely reflects clinical and methodological differences between studies. Age (infants vs. adolescents), severity of baseline pulmonary function, and infection status (initial vs. chronic infection by *P. aeruginosa* or *Burkholderia*) are factors that may modify the risk of AEs. The presence of comorbidities or colonization by multiple pathogens, as well as concomitant use of bronchodilators, mucolytics, azithromycin, and saline solutions, may influence both the incidence and the perception of adverse events. Dosing regimens also varied (75 mg TID for 14, 28, or 56 days) and may impact the accumulation of irritative effects. Follow-up duration and outcome definitions (for example, “dyspnea” versus “chest discomfort”) differed, contributing to the dispersion of results.

The present findings are largely consistent with eradication and long-term use studies from the past decade. The ALPINE2 study, a phase 3b trial comparing 14- versus 28-day regimens in 149 children with newly isolated *P. aeruginosa*, found similar eradication rates between groups (55.9% vs. 63.4%); treatment-emergent adverse events were similar, and no new safety signals were detected [31]. An earlier open-label study with 105 children confirmed that inhaled aztreonam was effective and well tolerated; 89.1% of patients were free of Pa at the end of treatment, and 58% remained negative at all subsequent assessments [22]. These results complement our meta-analysis by reinforcing that the tolerability of aztreonam is comparable between 14- and 28-day cycles. In adults, 2025 pharmacovigilance data based on the FAERS database identified new potential signals, such as cholestatic liver injury, hypoprothrombinemia, and hemoptysis, with a median onset of one year after starting AZLI [10]. Such events were rare and reported mainly in older adults, but highlight the need for liver monitoring, even in pediatric patients undergoing repeated or prolonged treatments.

Inhaled aztreonam achieves concentrations far above the MIC of *P. aeruginosa* directly within the airway lumen, providing minimal systemic exposure. This local administration explains the low frequency of systemic events (fatigue, fever, headache) and the absence of renal or hematological toxicity. On the other hand, deposition of the antibiotic in the airways can cause local irritation and induce bronchospasm or transient increases in sputum production. The observed increase in grade 3/4 respiratory disorders may reflect individual hypersensitivity, bronchial irritation, or interaction with thick secretions in patients with advanced lung disease. Laboratory abnormalities (hepatotoxicity) may be related to prolonged systemic absorption during repeated cycles, as suggested by pharmacovigilance signals in adults [10].

This review stands out for its comprehensive search strategy, which included multiple databases and grey literature, and for the independence of reviewers, reducing the risk of selection bias. The predominance of high-quality RCTs with similar dosing regimens (75 mg TID for 28 days) enabled the aggregation of comparable data. The inclusion of studies up to 2023, as well as consideration of long-term follow-up data, enhances the currency of the safety estimates.

### 4.3. Limitations

Several limitations must be acknowledged. Many estimates were derived from small samples and rare events, increasing imprecision and resulting in very high heterogeneity. There was variation in outcome definitions and in methods of event ascertainment (spontaneous reporting vs. standardized clinical evaluation), which hinders comparability. Most studies had a short duration (one 28-day cycle); data on prolonged and repeated use are scarce. Subgroup analysis (age, lung function, and initial vs. chronic infection) was not possible due to the lack of individual-level data, and selective publication of positive or safety-favorable RCTs cannot be excluded.

### 4.4. Certainty of Evidence (GRADE)

Applying the GRADE approach, the overall certainty for common respiratory events (productive sputum, congestion, dyspnea, fever) ranged from moderate to low, as RCTs provided precise estimates and the risk of bias was small; however, heterogeneity and imprecision reduced reliability. For rare or laboratory events (hepatotoxicity, hematological and renal abnormalities), certainty was very low, as estimates were based on single studies with few children. The main reasons for downgrading were risk of bias (unclear allocation concealment in some RCTs), imprecision (wide confidence intervals), inconsistency, and possible publication bias.

### 4.5. Implications for Clinical Practice and Future Research

The findings indicate that inhaled aztreonam can be administered relatively safely and is well-tolerated by children and adolescents with cystic fibrosis to eliminate or inhibit *Pseudomonas aeruginosa*, particularly when utilizing a regimen of 75 mg three times a day for 28 days. Monitoring should encompass an initial evaluation of respiratory function and vigilant observation for indications of bronchospasm or wheezing, especially in patients with severe pulmonary illness. Families and caregivers must be advised about the potential for moderate symptoms (sputum, weariness) and the infrequency of severe occurrences. For patients enduring numerous cycles or possessing risk factors for liver illness, laboratory evaluation of liver function and coagulation is advised, given the few indicators of cholestasis and hypoprothrombinemia observed in adult pharmacovigilance studies [10]. The choice to utilize should weigh the advantages of bacterial eradication or suppression and quality of life against the potential for severe respiratory exacerbations.

Subsequent investigations ought to incorporate randomized controlled trials (RCTs) including higher sample sizes and extended follow-up periods, proficient in identifying infrequent occurrences (hepatotoxicity, hematotoxicity, severe bronchospasm). The examination of various dosing schedules (e.g., 14 vs. 28 days) must persist to enhance adherence without jeopardizing efficacy or safety, as investigated in ALPINE2 [31]. Individual patient data meta-analyses and meta-regressions could investigate the observed heterogeneity by evaluating modifiers such as age, baseline lung function, infection type, and concurrent medications. Post-marketing safety studies and those utilizing extensive databases are crucial for detecting long-term uncommon signals, as indicated by pharmacovigilance analysis.

## 5. Conclusions

Inhaled aztreonam shows an acceptable safety and tolerability profile in children and adolescents with cystic fibrosis—adverse events are predominantly mild–moderate, reversible, and comparable to placebo, with no signal for excess mortality or hospitalization and a suggestion of preserved lung function. Nevertheless, the higher rate of grade 3/4 respiratory events and occasional laboratory abnormalities warrants close monitoring—especially with prolonged use—and supports the need for larger, longer, standardized studies to refine subgroup-specific risk. Meanwhile, clinicians should individualize therapy and monitor patients closely.

## Figures and Tables

**Table 1:** Characteristics and main findings of studies evaluating inhaled aztreonam safety and tolerability in children and adolescents.

Study	Period	Duration (mo)	Design	Countries	Clinical Characteristics	Adults Included	No of Participants (AZLI)	No of Female (AZLI)	AZLI Dose	AZLI Duration	Comparison	Concomitant Treatments	Study Purpose	Primary Outcome	Conclusions
Gilchrist (2023) [31]	2017–2021	25	RCT	EUR, USA	Children 3 mo–<18 y, CF, new-onset Pa infection	N	149	33	75 mg TID	14d or 28d per cycle; no retreatment	14d vs. 28d AZLI	NR**	Erad	Primary Pa eradication at 4 weeks after last AZLI dose	Non-inferiority of 14-day AZLI vs. 28-day AZLI not shown. Both courses well tolerated.
Keating (2021) [20]	2011–2016	60	OBS	USA	CF, ≥6 y, chronic Pa infection	Y	378	274	NR	NR	Cohort: AZLI use Y/N	NR**	Suppr	Proportion of subjects whose least susceptible *P. aeruginosa* isolate had an aztreonam MIC > 8 mg/mL and increased ≥4-fold from previous year; clinical outcomes included pulmonary exacerbations, hospitalizations, FEV1% predicted	Aztreonam susceptibility of *P. aeruginosa* remained stable over 5 years; reduced susceptibility was not associated with accelerated lung function decline, but with more exacerbations and hospitalizations.
Flume (2016) [21]	2012–2015	7	RCT	USA	CF, chronic Pa, ≥6 y, FEV1 25–75%, ≥1 IV abx past 12 mo	Y	43	24	75 mg TID	3 cycles × 28d (AZLI/TIS or placebo/TIS); total 84d (24w)	AZLI/TIS vs. PBO/TIS	Azithro, BCD, DNA, HTS, CCS	Treat chronic, prevent	Rate of protocol-defined pulmonary exacerbations (PDEs)	Continuous alternating inhaled regimen was well tolerated; AZLI/TIS may provide additional benefit vs. TIS alone.
Tiddens (2015) [22]	2011–2013	7	OBS	EUR, USA	Pediatric CF, 3 mo–<18 y, new Pa, FEV1 ≥ 80% (≥6 y), stable	N	105	58	75 mg TID	28d (single cycle, total)	–	Azithro, DNA, HTS	Erad/prevent	Percentage of patients with cultures negative for Pa at all post-treatment time points	AZLI was effective and well tolerated in eradicating Pa from newly infected pediatric patients with CF.
Tullis (2014) [23]	2010–2011	12	RCT	USA, CAN	CF, chronic Burkholderia infection	Y	48	22	75 mg TID	24w (continuous); up to 48w (extension)	AZLI vs. PBO	Azithro, DNA, HTS, abx	Treat chronic	Mean relative change from baseline FEV1% predicted (AUCave, weeks 0–24)	24-weeks of continuous AZLI did not significantly improve lung function in CF subjects with chronic Burkholderia spp. infection. AZLI was well tolerated.
Assael (2012) [24]	2008–2010	12	OBS	EUR, USA	CF, chronic Pa, FEV1 ≤ 75%	Y	136	68	75 mg TID	28d/cycle × 3 cycles (84d total)	AZLI vs. tobra	Azithro, DNA, HTS	Suppr/prevent	Change in FEV1% predicted (lung function); time to IV antibiotics	AZLI showed statistical superiority in lung function and reduced acute pulmonary exacerbations vs. TNS; both were well tolerated.
Wainwright (2011) [25]	2008–2009	1.4	RCT	USA, CAN, AUS	CF, Pa, FEV1 >75%, ≥6 y	Y	76	30	75 mg TID	28d/cycle	AZLI vs. PBO	DNA, azithro, PLP, BCD	Suppr/maint	Change from baseline at Day 28 on the CFQ-R Respiratory Symptoms Scale (CFQ-R RSS)	AZLI was well-tolerated. Effects on respiratory symptoms were modest; however, FEV1 improvements and bacterial density reductions support a possible role for AZLI in these relatively healthy patients.
Oermann (2010) [32]	2005–2008	18	OBS	USA, CAN, AUS, New Zealand	CF, chronic Pa airway infection	Y	274	123	75 mg BID or TID	28 d on/28d off × up to 9 courses (18 m)	75 mg BID vs. 75mg TID	Vits, PLP, BCD, DNA, CCS, azithro, HTS, tobra	Suppr/prevent	Safety and efficacy of repeated courses of inhaled aztreonam lysine (AZLI)	Repeated intermittent 28-day courses of AZLI were well tolerated, with clinical benefits in pulmonary function, quality of life, and weight; AZLI is a safe and effective new therapy in CF with PA infection.
Retsch-Bogart (2009) [26]	2005–2007	22	RCT	AUS, CAN, New Zealand, USA	CF, moderate-severe lung disease, Pa airway infection	Y	80	NR	75 mg TID	28d	AZLI vs. PBO	PLP, vits, BCD, DNA, CCS	Treat chronic	Change in patient-reported respiratory symptoms (CFQ-R Respiratory Scale)	28-day AZLI significantly improved respiratory symptoms and pulmonary function, and was well tolerated.
McCoy (2008) [9]	2005–2006	4	RCT	USA	CF, chronic Pa airway infection	Y	135	59	75 mg BID or TID	28d	AZLI vs. PBO (post-TIS run-in)	PLP, BCD, DNA, vits, azithro, CCS	Treat chronic/Prevent	Time to need for additional inhaled or intravenous antipseudomonal antibiotics	AZLI delayed time to need for antibiotics, improved symptoms and lung function, and was well tolerated.
Retsch-Bogart (2008) [27]	2003–2004	10	RCT	USA	CF, chronic Pa airway infection	Y	74	34	75 mg BID; 225 mg BID	14d BID	AZLI 75 mg vs. 225mg vs. PBO	BCD, CCS	Treat chronic	Percent change in FEV1 at Day 14	Trend toward increased respiratory symptoms in 225 mg group; reduction in *P. aeruginosa* density for both doses; no sig. difference in FEV1 at day 14 vs. placebo.
Gibson (2006) [28]	2003	0.43	RCT	USA	CF, stable, >12 y, FEV1 ≥ 40%	Y	23	11	75 mg, 150 mg, 225 mg (single dose)	3d (single daily dose); 3d follow-up	AZLI vs. PBO	BCD, AINES, CCS	Treat acute/Prevent	Safety, tolerability, microbiologic activity, and pharmacokinetics of inhaled aztreonam lysinate	Single doses of inhaled aztreonam lysinate up to 225 mg were safe and well-tolerated in adults and adolescents with CF; sputum concentrations exceeded MIC90 for *P. aeruginosa*; supports continued development.
Pryka (1987) [29]	NR	NR	RCT	USA	CF, pulmonary exacerbations, Gram-negative pneumonia	Y	11	6	75 mg TID	≥5d (mean 16.3d aztreonam; 15.7d control)	AZLI vs. azlocillin+tobra	Vits, PLP, theophylline, ranitidine, cimetidine, phenytoin, vitK, diflunisal, oxandrolone, terbutaline, metaproterenol, anti-staph (diclox/nafcillin)	Treat acute	Efficacy and safety of aztreonam versus azlocillin plus tobramycin in pulmonary exacerbations of CF	Aztreonam as effective as traditional therapy; transient liver enzyme elevation observed.
Tiddens (2015) [30]	NR	2	Post-hoc of RCT	NR	Children, CF, 3 mo–<18 y, new Pa infection	N	99	NR	75 mg TID	28d/cycle; 1 cycle	–	NR**	Erad/prevent	Factors associated with failure to eradicate or regrowth of PA post-AZLI	No baseline factors predicted Pa eradication failure after 28d AZLI; high compliance did not prevent failures; further studies needed.

Notes: Abx: antibiotics; AINES: non-steroidal anti-inflammatory drugs; AZLI: aztreonam lysine for inhalation; Azithro: azithromycin; BCD: bronchodilators; BID: twice daily; CCS: corticosteroids; CF: cystic fibrosis; CFQ-R: Cystic Fibrosis Questionnaire–Revised (Respiratory Symptoms Scale); d: days; DNA: dornase alfa; Erad: eradication of infection; FEV1: forced expiratory volume in 1 s; HTS: hypertonic saline; IV: intravenous; mo: months; NR: not reported; OBS: observational study; Pa: Pseudomonas aeruginosa; PBO: placebo; PLP: pancrelipase; Prevent: prevention of exacerbations; RCT: randomized clinical trial; Suppr: suppression of infection; TID: three times daily; TIS: tobramycin inhalation solution; Tobra: tobramycin; Vits: vitamins; w: weeks. Doses are presented as milligrams (mg) per administration and frequency (BID = twice daily; TID = three times daily). Duration is specified per treatment cycle and/or total study period as reported. “NR**” denotes data not reported by the study authors. “Adults inclusion” (Y/N): Indicates whether adults were included in the study population. “Primary Outcome” and “Conclusions” have been summarized for space; see full text for study-specific definitions and statistical methods. “Comparison” details the intervention group versus the control or comparator. “Concomitant Treatments” lists additional therapies administered alongside AZLI. Some studies included more than one arm or multiple dosing regimens; these are detailed in the respective rows.

**Table 2:** Summary of comparative risks for adverse effects associated with inhaled aztreonam treatments in children and adolescents: results from comparative meta-analysis.

Adverse Events	Assumed Risk of Comparator Group	Corresponding Risk with Aztreonam (95% CI)	Relative Effect RR (95%CI)	N° of Participants (N° of Studies)	Heterogeneity (I^2^, Q, *p*)	Quality of Evidence (GRADE)
Productive sputum	171 per 1000	141 per 1000 (114–174)	0.83 (0.67–1.02)	1.075 (7)	2.3%, *p* = 0.41, Q = 6.14	Moderate (1, 2)
Sputum increased congestion	74 per 1000	62 per 1000 (40–97)	0.84 (0.54–1.31)	806 (5)	0%, *p* = 0.41, Q = 3.95	Moderate (1, 2)
Cough	38.3 per 1000	37 per 1000 (33.2–42.1)	0.978 (95% CI 0.868–1.101)	496 (7)	0%, *p* = 0.6373 Q = 4.292	Low (1, 2, 3)
Fatigue	108 per 1000	76 per 1000 (41–141)	0.71 (0.38–1.31)	800 (4)	0%, *p* = 0.46, Q = 2.58	Moderate (1, 2, 6)
Dyspnea	184 per 1000	141 per 1000 (95–209)	0.76 (0.51–1.13)	733 (4)	0%, *p* = 0.59, Q = 1.93	Moderate (1, 2, 6)
Alterations in Oxyhemoglobin Saturation	59 per 1000	486 per 1000 (359–658)	0.83 (0.61–1.12)	459 (4)	0%, *p* = 0.71, Q = 1.39	Moderate (1, 2, 6)
Hemoptyse	92 per 1000	107 per 1000 (31– 364)	1.16 (0.34–3.95)	643 (3)	32%, *p* = 0.23, Q = 2.94	Low (1, 2, 6)
Crackles or rales	83 per 1000	70 per 1000 (50–100)	0.84 (0.60–1.20)	512 (3)	0%, *p* = 0.85, Q = 0.33	Moderate (1, 2, 6)
Chest pain or discomfort	24 per 1000	29 per 1000 (143–593)	1.22 (0.60–2.49)	617 (4)	0%, *p* = 0.66, Q = 1.61	Low (1, 2, 6)
Bronchospasm or wheezing	11 per 1000	12 per 1000 (5–36)	1.07 (0.46–2.48)	823 (5)	I^2^ = 35.7%; Q = 6.22; *p* = 0.18	Low(1, 2, 6)
Grade 3/4 respiratory disorders	83 per 1000	138 per 1000 (89–214)	**1.65 (1.07–2.57)**	720 (5)	0%, Q = 2.13, *p* = 0.71	Low (1, 2, 3)
Decreased Lung Function (PFT)	49 per 1000	34 per 1000 (26.6–43.9)	**0.70 (0.54–0.90)**	902 (6)	0% (*p* = 0.98; Q = 0.75)	Moderate: 1, 2, 3, 4
Pyrexia	56 per 1000	62 per 1000 (46–84)	1.11 (0.82–1.50)	927 (6)	0%, *p* = 0.77, Q = 2.54	Low (1, 2, 3)
Rhinorrhea, rhinitis, congestion, or sinusitis	101 per 1000	78 per 1000 (56–110)	0.78 (0.55–1.09)	985 (6)	I^2^ = 0%, *p* = 0.65, Q = 3.31	Low (1, 2)
Pharyngolaryngeal irritation	102 per 1000	103 per 1000 (69–139)	1.01 (0.70–1.46)	985 (6)	0%, *p* = 0.46, Q = 4.64	Low (1, 2, 3)
Abdominal pain or tenderness	71 per 1000	44 per 1000 (10 a 18,8)	0.62 (0.15 a 2.63)	880 (5)	I^2^ = 63%, *p* = 0.028, Q = 10.85	Very low (1, 2, 3)
Vomiting	106 per 1000	103 per 1000 (57–158)	0.97 (0.48 a 1.96)	268 (1)	–*	Very low (1, 2, 3, 4)
Dysgeusia	0 per 1000	68 per 1000 (51–108)	4.65 (0.27–81.62)	105 (1)	–*	Very low (1, 2, 3, 4, 6)
Decreased appetite	105 per 1000	92 per 1000 (6–140)	0.87 (0.06–13.37)	479 (2)	I^2^ = 0%, Q = 0.63, *p* = 0.43	Very Low (1, 2, 3, 6)
Allergic reactions	0 per 1000	0 per 1000 (0–0)	0.63 (0.11–3.63)	343 (3)	I^2^ = 0%, *p* = 0.862, Q = 0.30	Very Low (1, 2, 5, 6)
Headache	101 per 1000	104 per 1000 (71–154)	1.03 (0.70–1.52)	985 (6)	I^2^ = 0%, *p* = 0.567, Q = 3.88	Low (1, 2, 4)
Laboratory abnormalities	364 per 1000	727 per 1000 (309–1000)	2.00 (0.85–4.73)	22 (1)	–*	Very Low (1, 2, 3, 4, 6)
Hepatic abnormalities	182 per 1000	727 per 1000 (197–1000)	**4.00 (1.08–14.75)**	22 (1)	–*	Very Low (1, 2, 3, 4, 6)
Hematological abnormalities	0 per 1000	0 per 1000 (0–0)	1.00 (0.02–46.24)	22 (1)	–*	Very Low (1, 2, 3, 4, 5, 6)
Renal abnormalities	0 per 1000	0 per 1000 (0–0)	1.00 (0.02–46.24)	22 (1)	–*	Very Low (1, 2, 3, 4, 5, 6)
Serious adverse events (SAE)	83 per 1000	95 per 1000 (57–158)	1.14 (0.68–1.90)	1197 (9)	I^2^ = 32.3%, *p* = 0.16, Q = 11.81	Low (1, 2)
Hospitalizations	37 per 1000	33 per 1000 (20–53)	0.88 (0.54–1.44)	1002 (7)	I^2^ = 18.4%, *p* = 0.29, Q = 7.36	Low (1, 2, 3)
Deaths	0 per 1000	0 per 1000 (0–0)	1.27 (0.52–3.10)	1197 (9)	I^2^ = 0%, *p* = 0.93, Q = 3.12	Very Low (1, 2, 5, 6)
Withdrawal due to adverse events	21 per 1000	25 per 1000 (9.6–65.8)	1.18 (0.45–3.09)	1197 (9)	32.5%, *p* = 0.16, Q = 11.86	Very Low (1, 2, 6)
Diarrhea	111 per 1000	39 per 1000 (23.2–49.5)	0.36 (0.10–1.26)	157 (1)	–*	Very Low (1, 2, 6, 4)

**Abbreviations and Notes: CI**: confidence interval; **GRADE**: Grading of Recommendations, Assessment, Development, and Evaluations; **I^2^**: percentage of heterogeneity; **PFT**: pulmonary function test; **Q**: Cochran’s Q statistic for heterogeneity; **RR**: relative risk; **SAE**: serious adverse event. All risks are presented per 1,000 patients. “Assumed Risk of Comparator Group” refers to the event rate observed in control or comparison arms. “Corresponding Risk with Aztreonam (95% CI)” refers to the event rate in the aztreonam arm, with 95% confidence intervals. “Relative Effect” is expressed as relative risk (RR) with its 95% confidence interval. “GRADE” reflects the certainty of the evidence and reasons for downgrading (1: risk of bias, 2: imprecision, 3: indirectness/applicability, 4: incomplete data, 5: no numerical values, 6: very wide confidence interval). “–*” indicates the outcome was reported by only one study; therefore, meta-analysis and heterogeneity could not be assessed. For outcomes with zero events in either arm, the RR is estimated based on available data; however, interpretation should be cautious. “Statistically significant” results are highlighted in bold.

## Abbreviations

The following abbreviations are used in this manuscript:

AE	Adverse Event(s)
ALPINE2	Aztreonam Lysine for Pseudomonas Infection Eradication 2 (trial name)
Altera^®^	Altera Nebulizer System
AZLI	Aztreonam Lysine for Inhalation
BID	Bis in Die
CENTRAL	Cochrane Central Register of Controlled Trials
CF	Cystic Fibrosis
CFF	Cystic Fibrosis Foundation
CI	Confidence Interval
Clopper–Pearson	Clopper–Pearson Exact Method
CRD	Centre for Reviews and Dissemination
DRESS	Drug Reaction with Eosinophilia and Systemic Symptoms
eFlow^®^	eFlow Nebulizer System
FAERS	FDA Adverse Event Reporting System
FDA	Food and Drug Administration
FEV1	Forced Expiratory Volume in 1 Second
GRADE	Grading of Recommendations Assessment, Development and Evaluation
HKSJ	Hartung–Knapp–Sidik–Jonkman
I^2^	I-squared statistic
MEDLINE	Medical Literature Analysis and Retrieval System Online
MeSH	Medical Subject Headings
MIC	Minimum Inhibitory Concentration
NICE	National Institute for Health and Care Excellence
NLM	National Library of Medicine
*P. aeruginosa*	Pseudomonas aeruginosa
Pa	Pseudomonas aeruginosa
PFT	Pulmonary Function Test(s)
PICOS	Population, Intervention, Comparator, Outcomes, Study design
PRISMA	Preferred Reporting Items for Systematic Reviews and Meta-Analyses
PROSPERO	International Prospective Register of Systematic Reviews
R	R Programming Language
RCT	Randomized Controlled Trial
REML	Restricted Maximum Likelihood
RoB 2	Risk of Bias 2
RR	Relative Risk
RStudio	RStudio IDE
SAE	Serious Adverse Event(s)
TID	Ter in Die
U.S.	United States
τ^2^	Tau-squared

## Appendix A

**Table A1:** Risk of bias for studies of inhaled aztreonam in children and adolescents.

Study	Domain 1	Domain 2.1	Domain 2.2	Domain 3	Domain 4	Domain 5	Overall Judgment (RoB2)
Gilchrist (2023) [31]	L	L	L	S	L	S	S
Keating (2021) [20]	S	**S**	H	H	S	S	H
Flume (2016) [21]	L	**L**	L	L	L	S	S
Tiddens (2015) [22]	H	**S**	H	H	S	S	H
Tullis (2014) [23]	L	**L**	L	L	L	S	S
Assael (2012) [24]	L	**S**	H	S	S	S	H
Wainwright (2011) [25]	L	**L**	L	L	H	S	H
Oermann (2010) [32]	S	**L**	H	S	S	S	H
Retsch-Bogart (2009) [26]	L	**L**	H	L	L	S	H
McCoy (2008) [9]	L	**L**	H	L	L	S	H
Retsch-Bogart (2008) [27]	L	L	L	L	S	S	S
Gibson (2006) [28]	L	**L**	L	L	H	S	H
Pryka (1987) [29]	S	**S**	H	H	H	S	H
Tiddens (2015) [30]	S	**S**	H	L	H	S	H

Abbreviations and Notes: H: High risk of bias; L: Low risk of bias; S: Some concerns; RoB2: Risk Of Bias 2 tool. Domain 1: Randomization process; Domain 2.1: Deviations from intended interventions (effect of assignment); Domain 2.2: Deviations from intended interventions (effect of adherence); Domain 3: Missing outcome data; Domain 4: Measurement of the outcome; Domain 5: Selection of the reported result. All judgments are based on the RoB2 criteria. “Overall Judgment (RoB2)” reflects the highest risk rating across all domains for each study. For detailed criteria, see RoB2 guidance. Colors denote risk level: green (L) for low risk, yellow (S) for some concerns, and red (H) for high risk.

**Table A2:** Cumulative pooled prevalence of adverse events associated with inhaled aztreonam in children and adolescents: results from a proportional meta-analysis.

Adverse Events	Pooled Prevalence % (95% CI)	No of Participants (Studies)	Heterogeneity (I^2^, Q, *p*)	Quality of the Evidence (GRADE)
Hemoptysis	0.15 [0.01–0.43]	625 (4)	I^2^ = 95.4%, *p* < 0.0001, Q = 64.77	Very low ^1,2,3,6^
Productive sputum	0.25 [0.08–0.47]	960 (9)	I^2^ = 98.2%, *p* < 0.0001, Q = 455.92	Very low ^1,2,3,6,7^
Sputum increased congestion	0.19 [0.03–0.43]	732 (6)	I^2^ = 97.0%, *p* < 0.0001, Q = 166.46	Very low ^1,2,6^
Fatigue	0.13 [0.01–0.34]	701 (5)	I^2^ = 96.6%, *p* < 0.0001, Q = 118.97	Low ^1,2,5^
Dyspnea	0.14 [0.03–0.32]	773 (6)	I^2^ = 95.4%, *p* < 0.0001, Q = 109.80	Low ^1,2,6^
Acute Respiratory Infections	0.10 [0.00–0.45]	319 (5)	I^2^ = 95.7%, *p* < 0.0001, Q = 93.90	Low ^1,2,6^
Influenza	0.01 [0.01–0.02]	150 (2)	I^2^ = 0.0%, *p* = 0.98, Q = 0.00	Low ^1,2,3,4^
Alterations in Oxyhemoglobin Saturation	0.0 [0.0–2.3]	74 (1)	–*	Very low ^1,2,3,5^
Crackles or rales	0.14 [0.02–0.33]	558 (4)	I^2^ = 88.8%, *p* < 0.0001, Q = 26.80	Low ^1,2,3^
Chest pain or discomfort	0.11 [0.04–0.21]	632 (5)	I^2^ = 86.4%, *p* < 0.0001, Q = 29.34	Low ^1,2,6^
Bronchospasm or wheezing	0.10 [0.02–0.21]	815 (7)	I^2^ = 93.3%, *p* < 0.0001, Q = 89.03	Low ^1,2,3,5^
Grade 3/4 respiratory disorders	0.15 [0.06–0.27]	992 (7)	I^2^ = 94.7%, *p* < 0.0001, Q = 113.00	Low ^1,2,5^
Decreased pulmonary function (PFT)	0.06 [0.02–0.11]	959 (9)	I^2^ = 87.3%, *p* < 0.0001, Q = 63.10	Low ^1,2,6^
Lung transplant	0.00 (0.00–0.0010)	291 (3)	I^2^ = 0.0%, *p* = 0.97, Q = 0.05	Very low ^1,2,4,5,6^
Fever or pyrexia	10.9 [2.4–23.7]	965 (9)	I^2^ = 96.1%, *p* < 0.0001, Q = 206.50	Low ^1,2,4,5,6^
Rhinorrhea, rhinitis, congestion, or sinusitis	0.13 [0.08–0.19]	1022 (9)	I^2^ = 87.2%, *p* < 0.0001, Q = 62.46	Low ^1,2,6^
Pharyngolaryngeal irritation	0.16 [0.05–0.31]	843 (7)	I^2^ = 95.9%, *p* < 0.0001, Q = 144.70	Low ^1,2,3,6^
Abdominal pain or tenderness	4 [1–11]	495 (5)	I^2^ = 75.0%, *p* = 0.003, Q = 16.02	Low ^1,2,3,6^
Vomiting	5 [3–8]	241 (2)	I^2^ = 90.8%, *p* = 0.001, Q = 10.88	Very low ^1,2,3,4,6^
Dysgeusia	0.07 [0.02–0.15]	74 (1)	–*	Very low ^1,2,5,6^
Decreased appetite	0.17 [0.00–0.75]	545 (3)	I^2^ = 97.9%, *p* < 0.0001, Q = 96.30	Low ^1,2,6^
Allergic reactions	0.00 [0.00–0.50]	167 (3)	I^2^ = 0.0%, *p* = 0.84, Q = 0.35	Low ^1,2,4,5^
Headache	0.13 [0.05–0.24]	843 (7)	I^2^ = 92.3%, *p* < 0.0001, Q = 78.43	Low ^1,2,6^
Audiological abnormalities	1.7 [0.0–9.4]	354 (4)	I^2^ = 75.2%, *p* = 0.007, Q = 12.12	Low ^1,2,6^
Hepatic abnormalities	0.23 [0.00–1.00]	146 (2)	I^2^ = 97.4%, *p* < 0.0001, Q = 38.94	Very low ^1,2,4,5,6^
Hematological abnormalities	0.00 [0.00–0.15]	11 (1)	–*	Very low ^1,2,4,5,6^
Renal abnormalities	0.00 [0.00–0.14]	146 (2)	I^2^ = 0.0%, *p* = 0.50, Q = 0.45	Very low ^1,2,4,5,6^
Laboratory abnormalities	1.7 [0.0–9.4]	354 (4)	I^2^ = 75.2%, *p* = 0.007, Q = 12.12	Low ^1,2,6^
Serious adverse events (SAE)	0.11 [0.03–0.23]	1124 (12)	I^2^ = 96.1%, *p* < 0.0001, Q = 285.70	Low ^1,2,3,6^
Hospitalizations	15.3 [4.9–29.7]	1.517 (11)	I^2^ = 97.3%, *p* < 0.0001, Q = 376.70	Low ^1,2,3,6^
**Deaths**	0.02 [0.00–0.36]	1124 (12)	I^2^ = 0.0%, *p* = 0.72, Q = 7.95	Low ^1,2,3,4^
Withdrawal due to adverse events	0.03 [0.01–0.05]	1.634 (13)	I^2^ = 80.9%, *p* < 0.0001, Q = 62.95	Low ^1,2,6^
Missed dose (“Dose Missed”)	0.01 [0.00–0.05]	105 (1)	–*	Very low ^1,2,5,6^
Diarrhea	0.05 [0.02–0.10]	150 (2)	I^2^ = 0.0%, *p* = 0.46, Q = 0.54	Low ^1,2,5^

Abbreviations and Notes: CI: Confidence interval; GRADE: Grading of Recommendations, Assessment, Development and Evaluation; I^2^: Percentage of statistical heterogeneity; *n*: Number; PFT: Pulmonary function test; Q: Cochran’s heterogeneity statistic; RR: Relative risk; SAE: Serious adverse event. All prevalence estimates are pooled proportions calculated using a random-effects model and are presented as percentages with corresponding 95% confidence intervals (CIs). Heterogeneity is reported as I^2^ (percentage of total variation due to heterogeneity), Cochran’s Q, and *p*-value. GRADE quality assessments were downgraded for the following reasons: 1 = risk of bias; 2 = imprecision; 3 = applicability concerns; 4 = incomplete data; 5 = lack of numerical values; 6 = wide confidence interval. “–*” indicates the outcome was reported in only one study. Outcomes with a pooled prevalence of less than 1% are considered rare events. Deaths, serious adverse events (SAEs), and withdrawals are presented as proportions of the total cohort. Full statistical methods are detailed in the Methods section.
